# Understorey *Rhododendron tomentosum* and Leaf Trichome Density Affect Mountain Birch VOC Emissions in the Subarctic

**DOI:** 10.1038/s41598-018-31084-3

**Published:** 2018-09-05

**Authors:** Adedayo O. Mofikoya, Kazumi Miura, Rajendra P. Ghimire, James D. Blande, Minna Kivimäenpää, Toini Holopainen, Jarmo K. Holopainen

**Affiliations:** 1 Department of Environmental and Biological Sciences, University of Eastern Finland, P. O. Box 1627, 70211 Kuopio, Finland; 2 Institute of Biology, Freie Universität Berlin, Haderslebener Str.9, 12163 Berlin, Germany

## Abstract

Subarctic vegetation is composed of mountain birch [*Betula pubescens ssp*. *czerepanovii* (MB)] forests with shrubs and other species growing in the understorey. The effects of the presence and density of one understorey shrub, *Rhododendron tomentosum* (RT), on the volatile emissions of MB, were investigated in a Finnish subarctic forest site in early and late growing season. Only MB trees with an RT-understorey emitted the RT-specific sesquiterpenoids, palustrol, ledol and aromadendrene. Myrcene, which is the most abundant RT-monoterpene was also emitted in higher quantities by MB trees with an RT-understorey. The effect of RT understorey density on the recovery of RT compounds from MB branches was evident only during the late season when sampling temperature, as well as RT emissions, were higher. MB sesquiterpene and total emission rates decreased from early season to late season, while monoterpene emission rate increased. Both RT and MB terpenoid emission rates were linked to density of foliar glandular trichomes, which deteriorated over the season on MB leaves and emerged with new leaves in the late season in RT. We show that sesquiterpene and monoterpene compounds emitted by understorey vegetation are adsorbed and re-released by MB, strongly affecting the MB volatile emission profile.

## Introduction

Subarctic vegetation is dominated by mountain birch forests with dwarf shrubs growing in the understorey^1,2^. The subarctic region is marked by harsh environmental conditions and shortage of nutrients, which limits plant growth. Therefore, subarctic vegetation is composed of dwarf or miniature versions of the same species found in warmer climates^3^. Apart from physical adaptation, plants use chemical means such as the emission of volatile organic compounds (VOCs) to adapt and acclimate to the environmental conditions in their habitat^4^. These emissions are dependent on abiotic factors especially temperature and light^4^, which have characteristic fluctuations in the arctic regions. Due to low temperature, low foliar density and solar angle, early global VOC emission models assigned minimal emissions to polar regions^3,5^. However, a number of field measurements from the region have shown VOC emissions and emission potentials from subarctic regions to be substantial and sometimes higher than emissions from boreal forests^6–8^. These high emissions may be due to the large temperature fluctuations, differences between air and leaf surface temperature as well as long periods of light during the growing season^3,9,10^.

Plant-emitted VOCs mediate a number of ecological interactions including plant-to-plant interactions^11^, where VOCs emitted by a plant may affect neighbouring plants via passive or active means. Active plant-to-plant interactions result in a physiological change in the receiving plants mediated by VOCs released from a focal plant^12,13^. Passive interactions on the other hand, involve VOCs adsorbing to the surfaces of neighbouring plants, which may improve resistance to herbivores in the receiver plant^14,15^. Passive adsorption and re-release of neighbouring plant VOCs has been reported to increase resistance to herbivores in boreal ecosystems^14^ and is dependent on the distance between the emitter and receiver plant and the air temperature^14,16^.

The vertical distances between tree canopies and understorey shrubs in subarctic ecosystems are relatively short compared to boreal ecosystems due to the trees being dwarf species. The mountain birch, *Betula pubescens ssp*. *czerepanovii* (N. I. Orlova) Hämet-Ahti (henceforth referred to as MB), and the shrub, *Rhododendron tomentosum* Harmaja (henceforth referred to as RT), are common co-existing species of the Finnish subarctic. MB trees are introgressive hybrids between the diploid (n = 28) dwarf (*Betula nana* L.) and the tetraploid (n = 56) downy (*Betula pubescens* Ehrh.) birch^17,18^. Young MB leaves are covered with glandular trichomes^19^, which are specialized structures that serve in the storage of VOCs and other plant secondary metabolites in a number of plants^20–22^. In *Betula spp*, sesquiterpene emission rates have been linked with glandular trichome density^23^.

**Figure 1:**
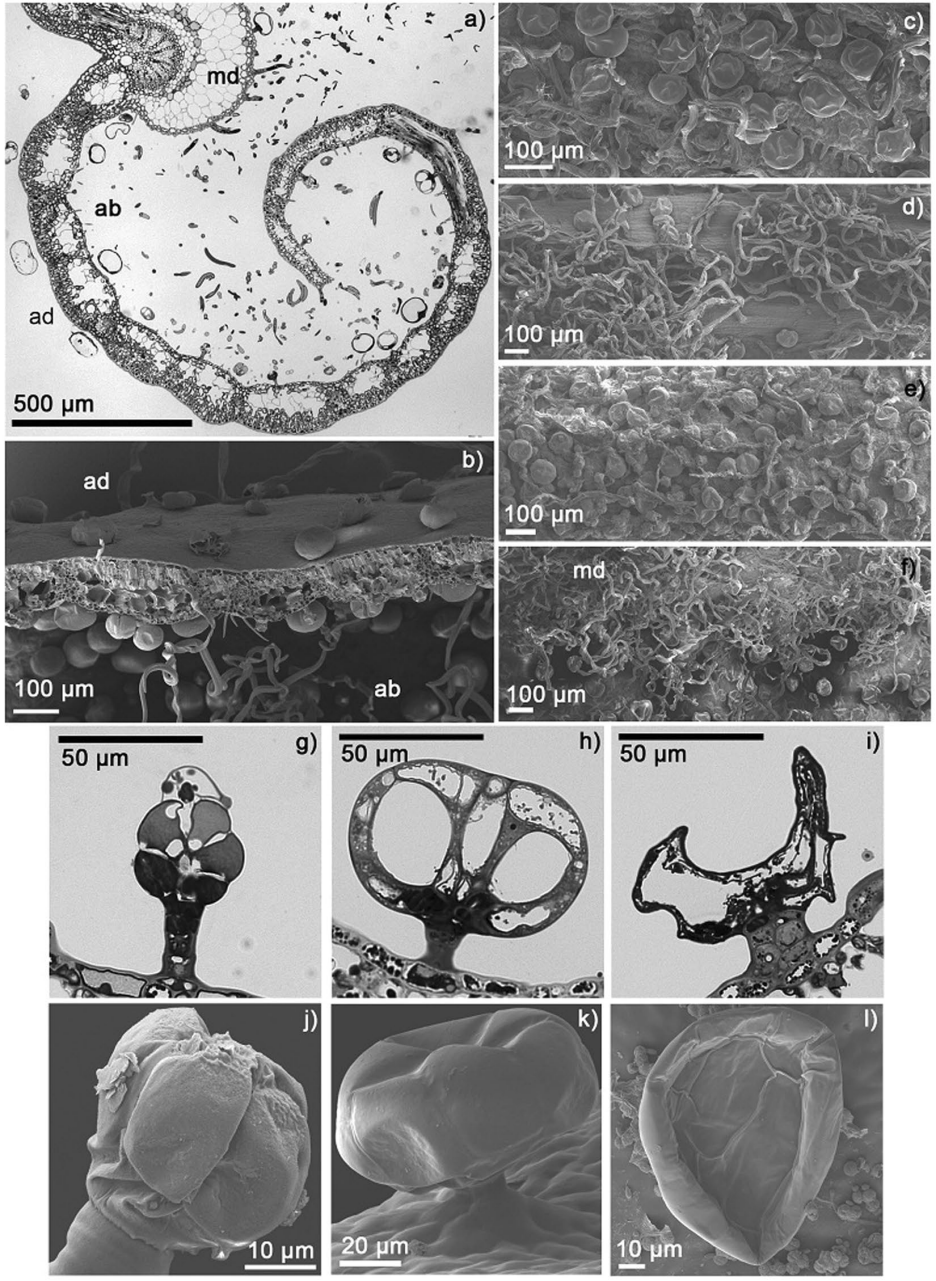
Light microscopy (LM) and scanning electron microscopy (SEM) images of trichomes of *Rhododendron tomentosum*. (**a**) LM cross-section of a leaf half with glandular trichomes on abaxial (ab) and adaxial (ad) sides. Cross-sections of long, hairy trichomes appear in a space on the abaxial side. (**b**) SEM sample of fractured leaf showing spherical, glandular trichomes on the abaxial and adaxial leaf sides and long, hairy trichomes. (**c**) Glandular, long hairy and short hairy trichomes on bud surface. SEM. (**d**) Glandular and long hairy trichomes on surface of stem and (**e**) petiole of older leaf. SEM. (**f**) Abundant long, hairy trichomes on the midrib (md), and glandular trichomes on the adaxial leaf surface. SEM. (**g–l**) Details of glandular trichomes with stalk and multicelled head in (**g–i**) LM and (**j–l**) SEM images. (**g** and **j**) developing glandular trichome, (**h** and **k**) full sized intact glandular trichome, (**i** and **l**) collapsed, deteriorated trichome. Note particle accumulation in (**l**). Particles of abiotic and biotic origin typically accumulated on and around deteriorated trichomes on the adaxial surface of leaves.

RT is a woody perennial evergreen shrub distributed throughout boreal and subarctic ecosystems^14^. We observed that it is characterized by a dense distribution of glandular trichomes on stem and leaf surfaces (Fig. 1), and has a characteristic smell resulting from a high terpenoid content^24^. The terpenes of RT include the arthropod-repelling^25^ C15 semi-volatile sesquiterpenoids ledene (C_15_H_24_), ledol (C_15_H_26_O), and palustrol (C_15_H_26_O)^26^. In addition, different RT provenances emit large amounts of myrcene as a major monoterpene compound^14,24,27^. Myrcene is a highly reactive monoterpene compound – reacting with ozone and OH radicals to form reaction products such as terpenylic acid in the atmosphere^28^. Ledol and palustrol are oxygenated sesquiterpenes that are not readily degraded in the atmosphere^29^, they also have semi-volatile characteristics, and as such may persist on leaf surfaces for long periods^30^.

Understorey vegetation is an abundant source of VOC emissions in the subarctic^2^, which may in turn mediate passive or active interactions with tree stands growing above them. RT-specific compounds have been recovered from the VOC emissions of neighbouring plants in boreal ecosystems and laboratory experiments^14,16^. The adsorption and re-release of RT sesquiterpenes by neighbouring plants has been shown to be dependent on temperature^16^. The temperature dependence of plant terpenoid emissions^4^ as well as adsorption and re-release of VOCs by neighbouring plants^16^ makes studying the process in the subarctic relevant. In light of a changing climate, arctic regions are most at risk, with models predicting an up to 4 °C temperature increase for arctic summers^31^. Furthermore, subarctic vegetation is sensitive to temperature changes, for example, a 2 °C increase in temperature doubled terpenoid emissions from heath vegetation in the Swedish subarctic^32^. These temperature changes may also result in a change in plant species composition and subsequently the quality and quantity of VOC emission profiles in the regions.

In this study, we investigated the effects of the presence and coverage of understorey RT shrubs on the VOC emissions of MB trees during early and late growing season. We also investigated the role of the condition and density of foliar glandular trichomes on VOC emissions from RT and MB. We hypothesised that (a) RT VOCs will stick to and be recovered from neighbouring MB branches. (b) Recovery of RT VOCs from MB is dependent on the density of RT growing in the understorey and (c) VOC emission rate from both RT and MB is dependent on glandular trichome density.

## Materials and Methods

### Vegetation assessment

**Figure 2:**
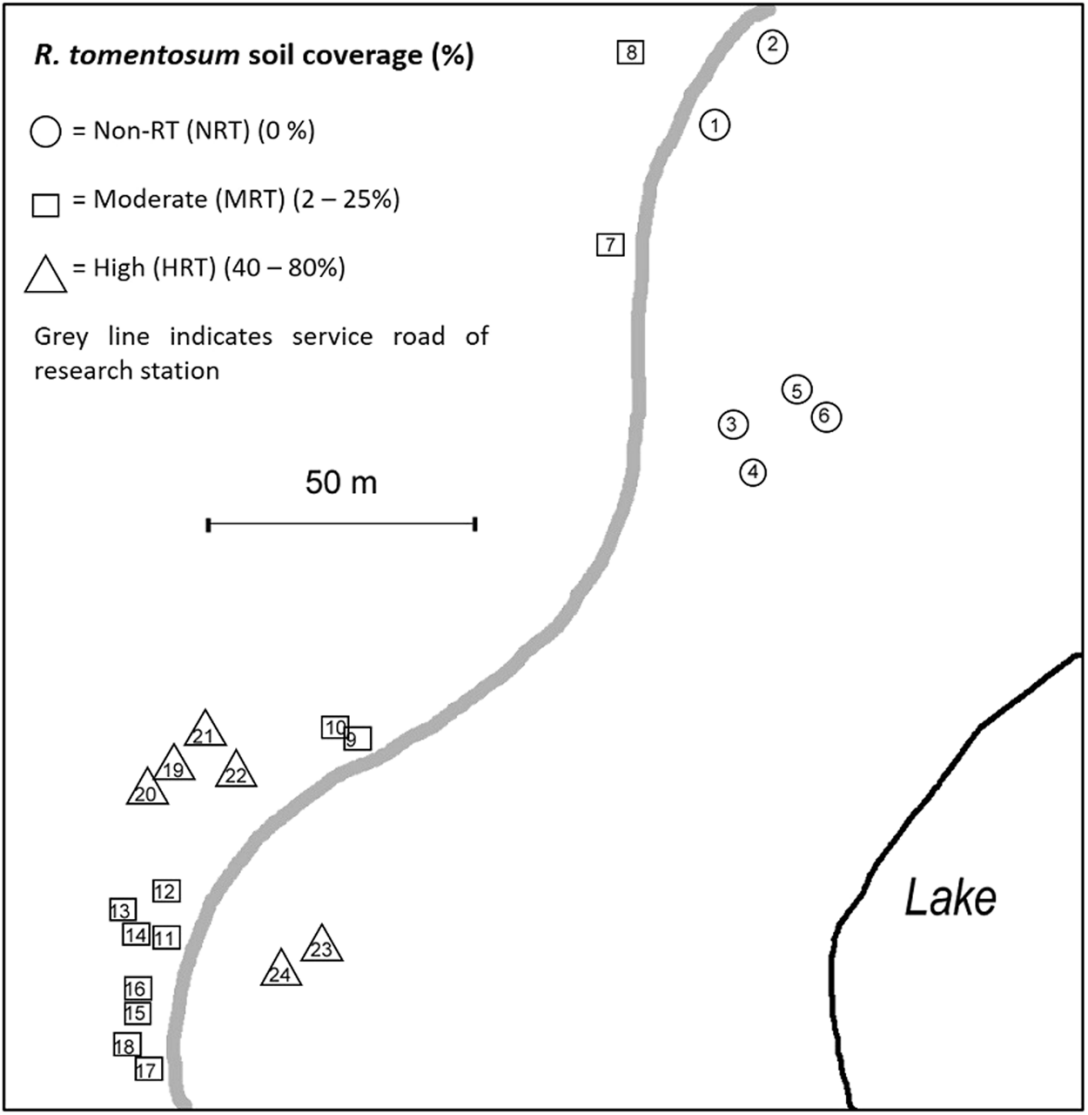
Schematic map showing sites of sampled *Betula pubescens* (MB) trees and *Rhododendron tomentosum* (RT) shoots. Site is located in the Kevo Subarctic Research Institute of the University of Turku in the upper Finnish Lapland (69°45′N, 27°01′E).

Field sampling campaigns were conducted in 2015 and 2017 at the Kevo Subarctic Research Institute of the University of Turku located in upper Finnish Lapland (69°45′N, 27°01′E) within the Kevo strict nature reserve area. The first sampling was done between 30th June and 2nd July 2015, the second sampling between 27th and 30th June 2017 and the 3rd (late season) sampling between 14th and 18th August 2017. During the first sampling, 24 mountain birch, *Betula pubescens ssp*. *czerepanovii* (N. I. Orlova) Hämet-Ahti (MB) trees were selected and grouped based on the density of *Rhododendron tomentosum* (RT) shoots in the understorey (Fig. 2). The Non-RT control group (NRT) (n = 6) had no understorey presence of RT shoots, the moderate RT group (MRT, n = 12) had 2–25% RT coverage in the understorey and the high RT group (HRT, n = 6) had 40–80% RT coverage in the understorey (Tables S1 and S2).

### VOC sampling

During the first sampling campaign, we selected a single branch from each of the 24 MB trees for VOC sampling. Under the canopy of each MB tree, we marked a 1 m^2^ area, and selected one branch that was situated directly above the area marked on the ground for VOC sampling. In subsequent campaigns, two branches were sampled for VOCs per tree. Also during the first sampling campaign, 10 RT branches (6 from HRT plots and 4 from MRT quadrants) were selected for VOC collection. In the 2nd and 3rd sampling campaigns, two RT branches were sampled per quadrant. In total, we collected 24 VOC samples from MB in 2015 and 48 samples for each sampling campaign of 2017, while for RT we collected 10 samples in 2015 and 36 samples for each sampling campaign in 2017.

VOC sampling was performed with the dynamic headspace sampling technique^33^. Each sampled branch was enclosed in a cooking bag (polyethylene terephthalate, 25 × 55 cm) that had been pre-heated in an oven (120 °C) for 1 h. Sampled MB branches contained 15–36 full-grown leaves. In June, the leaves were about 3 weeks old. An average of 54 leaves were present on sampled RT shoots, in June, shoots contained older leaves that developed in the previous growing season and in August, new leaves had begun to emerge at the tip of sampled shoots. A flexible wire was used to tie the bag at the base of the branch and a hole was made at the top corner of the bag. A Teflon tube was inserted through the hole and fastened in place. Ozone-free, activated carbon-filtered replacement air (airflow ~300 ml min^−1^) was passed through the Teflon tube and into the bags. After 10 minutes, in which time the air in the bags was exchanged, a Tenax TA adsorbent-filled tube was inserted through a hole made at the second top corner of the bag and connected to a suction tube that pulled air through the tube at a rate of ~200 ml min^−1^. All openings were made airtight by tightly closing them with a flexible wire. The collection time for VOC samples from the MB branches was 30 minutes after which sample tubes were sealed with brass caps and stored in a cold box until analysis. VOCs from RT shoots were collected in a similar way but with the collection time reduced to 10 minutes during the first sampling campaign and 20 minutes for subsequent samplings. Photos of the sampled MB branches were taken after each VOC collection for measurement of leaf area (LA). Sampled RT shoots were cut and dried at 60 °C for 3 days, dry weight (DW) was subsequently measured.

### VOC analysis

VOCs were analysed by gas chromatography-mass spectrometry (Hewlett Packard GC 6890, MSD 5973). The compounds adsorbed in the tube were desorbed in a thermal desorption unit at 250 °C for 10 minutes, cryofocused in a cold trap at −30 °C and injected into an HP-5MS capillary column (50 m × 0.2 mm i.d. × 0.5 µm; Agilent Technologies, USA), helium was the carrier gas. Oven temperature was at 40 °C for one minute, then raised to 210 °C at 5 °C min^−1^ and further to 250 °C at 20 °C min^−1^. Volatile compounds were identified by comparing their mass spectra, retention times and peak areas with those of pure standards and the Wiley library. In cases where standards were unavailable, compounds were quantified using α-pinene, 1,8-cineole and longifolene as reference compounds for non-oxygenated monoterpenes, oxygenated monoterpenes and sesquiterpenes, respectively. MB emission rates were expressed in ng LA cm^−2^ h^−1^ and RT emission rates in ng g^−1^ leaf DW h^−1^.

MB and RT emission rates were standardized relative to a temperature of 30 °C using the algorithm reported by Guenther *et al*.^34^, the temperature coefficient β was 0.17 for sesquiterpenes and 0.1 for monoterpenes and other compounds. Adhered compounds (RT compounds recovered from MB shoots) were not standardized because their emission is not related to MB photosynthetic activity. During VOC sampling, the temperature and humidity in the PET bag was measured with wireless temperature/humidity loggers (Hygrochron DS1923- F5 i-Button, Maxim Integrated Products, Inc., CA). Mean sampling temperatures were 15.1 °C in June 2015, 13.2 °C in June 2017 and 15.4 °C in August 2017, respectively.

### Trichome assessment

Scanning electron microscopy (SEM) was used to evaluate the density and condition of glandular trichomes on MB leaves and RT leaves and stems in early and late season of 2017. Samples were collected from two plants of each species from NRT, MRT and HRT sites, thus n = 6 for MB and n = 4 for RT for each sampling period. In June, two MB leaves of similar size and developmental stage as the shoot used for VOC collection were carefully detached from a ramet adjacent to the VOC collection ramet, and placed in paper bags. In August, three representative leaves were selected. RT shoots representative of the MRT and HRT sites were cut, and leaves and pieces of stem were attached to double-sided tape on the Petri dishes. Samples were dried and stored at room temperature before further analysis.

Two leaf segments sized ~3 × 3 mm were cut from the middle of the MB leaves next to the midrib. One segment was placed with the upper (adaxial) and the other segment lower (abaxial) side upwards on copper-coated tape on SEM stubs. For RT, 7 mm long leaf segments were cut from the middle part of 2–4 leaves per leaf age present (leaves that developed in 2015 and 2016 were collected in June; and leaves that developed in 2016 and 2017 were collected in August). In addition, samples were prepared from RT stems on both sampling dates. The samples were coated with an ~50 nm layer of gold (Agar Auto Sputter Coater B7341, Agar Scientific Ltd., Stansted, UK). Coated samples were stored in a desiccator prior to the SEM study.

The samples were examined using a High Resolution Scanning Electron microscope (HR-SEM; Carl Zeiss, Sigma HD|VP, Oberkochen, Germany). A 2.5 mm^2^ area from the MB sample was digitally photographed, and the glandular trichome density (# mm^−2^) was determined. In addition, the proportion of deteriorated glandular trichomes (where the glandular structure was worn or collapsed) was calculated. In RT, the glandular trichome density (# mm^−2^) on the adaxial surface was determined from the planar leaf areas from four images per leaf. Glandular trichome densities were also studied on RT stems. The proportion of deteriorated (glandular structure flattened) glandular trichomes on both leaves and stems were calculated. Quantitative image analyses were done using the tools of Image J ver. 1.47 v (http://imagej.nih.gov/ij/).

### Statistical analysis

All statistical analyses were performed using the SPSS statistics 23.0 package (SPSS, Inc, Chicago, IL, USA). One-way ANOVA with Tukey’s or Dunnet T3 post-hoc tests were used to test differences in VOC emission rates between the three MB groups. Shapiro Wilk test (normality of the data) and Levene’s test (homogeneity of variances) were used to confirm assumptions for analysis of variance. Data were log-transformed to fulfil the ANOVA assumptions and in cases where data did not meet assumptions, the non-parametric Kruskal-Wallis test with multiple pairwise comparison (Mann-Whitney U with Bonferroni corrections) was used. Differences in early and late season emission rates for MB in 2017 were tested with paired sample T-test and in RT, the Mann-Whitney U test was used to test differences in emission between groups and between sampling dates. Differences in glandular trichome density and condition between early and late season for MB leaves were studied by Mann-Whitney U test. The Spearman rank correlation test was used to test the relationship between the number of RT shoots in the understorey and adhered compound emissions from MB (only MRT and HRT plots included).

## Results

### Mountain birch (MB) shoot emissions

**Figure 3:**
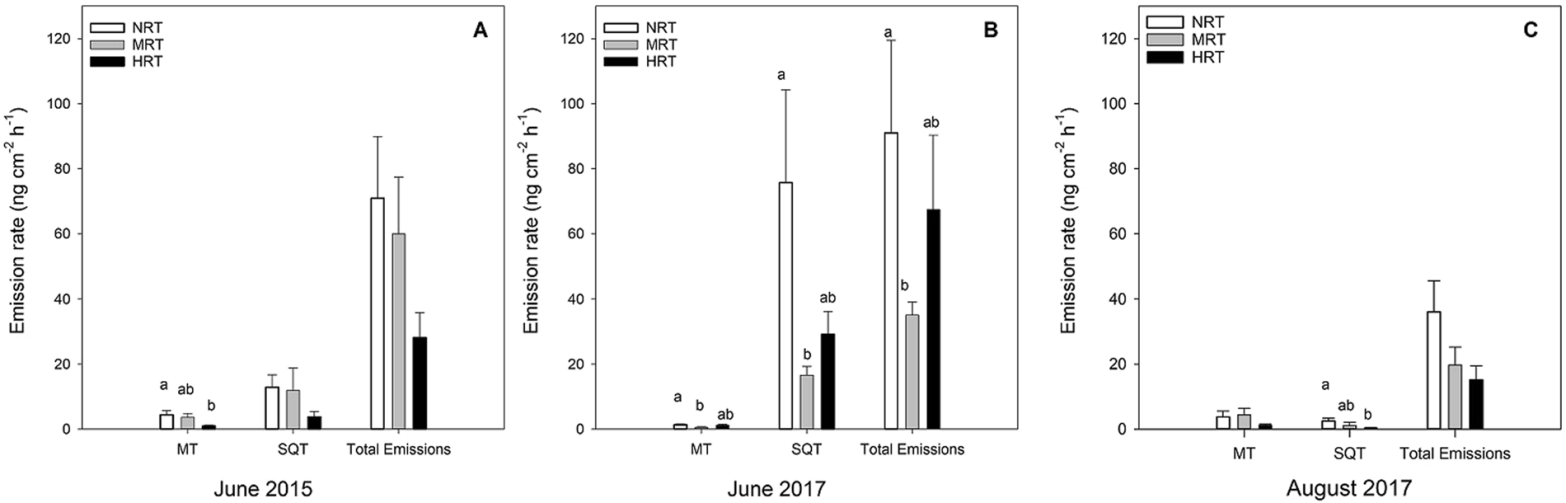
Emission rates (ng cm^−2^ h^−1^) (mean ± SE) of monoterpenes, sesquiterpenes, and total emissions from mountain birch (MB) branches with zero (NRT), moderate and high density of RT (MRT and HRT) shrubs growing in the understorey. Differences in emission rates between groups were tested with One-way ANOVA and Kruskal-Wallis non-parametric tests with multiple pairwise comparisons. Different letters above the bars indicate significant difference between groups (*P* < 0.05).

The total emission rate of monoterpenes differed between RT groups in June 2015 (one-way ANOVA, *F*_2,21_ = 3.874, *P* = 0.037) and June 2017 (*F*_2,21_ = 3.995, *P* = 0.034), being highest in NRT in both cases (Fig. 3A,B). From multiple comparisons, the MB monoterpene emission rate for NRT was higher than HRT in June 2015 and higher than MRT in June 2017. Total sesquiterpene emission rates were also significantly affected in June 2017 (one-way ANOVA, *F*_2,21_ = 9.108, *P* = 0.001) and August 2017 (Kruskal-Wallis H test, χ^2^(2) = 6.829, N = 24, *P* = 0.033) being highest in NRT groups (Fig. 3B,C). From multiple comparisons, the emission rate of sesquiterpenes was higher in NRT than in MRT in June 2017 and higher in NRT than HRT in August 2017. Total MB emissions were highest in the NRT group in June 2017 (one-way ANOVA, *F*_2,21_ = 4.574, *P* = 0.022), the same trend was observed in June 2015 and August 2017 (Fig. 3A,B). Mean emission rates of individual compounds are presented in the Supplementary data (Table S1).

### *R. tomentosum* (RT) emissions

The major VOCs emitted by RT shoots were the monoterpene, myrcene (40%), and the sesquiterpenoids, palustrol (23%), aromadendrene (13%), and ledol (4% of total emissions) (Table 1). There were no differences in emission rates of major RT compounds between MRT and HRT. However, the emission rates of 6-methyl-5-hepten-2-one was higher in MRT than HRT in June 17 and the emission rate of Δ-3-carene was higher in MRT in August 2017 (Table 1).

**Table 1:** Volatile organic compound emissions of *R*. *tomentosum* (RT) from moderate RT (MRT) density and high RT (HRT) groups in early (June) and late (August) season of 2017, median (inter quartile range, ng g^−1^ h^−1^). Differences between groups; MRT density and HRT density during both sampling occasions, and differences between June and August emissions from pooled MRT and HRT data were tested with the Mann-Whitney U test, (*P* < 0.05) emboldened.

	June 17				August 17				Pooled Data (June vs August N = 18)	
	MRT (N = 12)	HRT (N = 6)	U	*P*	MRT (N = 12)	HRT (N = 6)	U	*P*	U	*P*
**Monoterpenes**										
α–Pinene	166.04 (92.44–894.53)	63.28 (43.99–74.56)	12	0.064	312.11 (102.17–5134.85)	154.13 (135.15–374.4)	32	0.750	100	0.083
Camphene	93.13 (43.01–506.96)	37.07 (27.8–55.84)	17	0.195	126.98 (82.72–3933.87)	137.81(128.32–320.99)	32	0.750	87	**0.029**
Sabinene	135.49 (102.23–923.58)	81.68(34.08–154.62)	18	0.234	0 (0–6101.61)	0 (0–0)	29	0.553	78.5	**0.013**
β-Pinene	93.41(57.76–621.15)	43.13(19.51–58.79)	14	0.104	84.46 (40.95–2929.59)	87.85(43.91–216.18)	32	0.750	135.5	0.568
Myrcene	132199.54(60066–214656)	64062 (59484–90397)	26	0.721	174655(77244–234585)	196565(167787–285991)	28	0.494	103	0.103
Δ–3–Carene	6.89 (0–98.61)	0 (0–0)	13	0.064	75.75(24.82–382.05)	0 (0–21.25)	15	**0.050**	107	0.134
Terpinene	735.97 (233.25–9614.04)	269.63 (76.13–533)	19	0.279	101.02 (10.76–1102.12)	0 (0–107.21)	22	0.213	95.5	0.057
Cymene	174.14 (140.10–2964.79)	141.39 (65.31–196.12)	18	0.234	871.30 (549.91–48331.05)	1464.56 (771.78–4039.82)	32	0.750	46	<**0.001**
Limonene	206.33 (123.54–630.31)	82.31 (43.71–119.18)	13	0.082	465.66 (203.98–14714.09)	369.78 (261.89–828.88)	31	0.682	76	**0.010**
(Z)–β–Ocimene	1286 (432.52–1691)	591.09 (352.47–1197.77)	16	0.383	9718.68 (3707–23105)	7771.91 (6906.6–19665.54)	30	0.616	16	<**0.001**
(E)–β–Ocimene	682.75 (411.29–1969.02)	395.68 (205.75–544.23)	18	0.160	2391(1303.5–8605.3)	3601.72 (2658.7–6061.3)	28	0.494	38	<**0.001**
γ–Terpinene	176.09 (91.23–1885.44)	93.21 (35.32–139.29)	18	0.234	60.40 (27.91–2057.68)	62.23 (42.58–141.75)	35	0.964	121	0.303
α–Terpinolene	79.75 (52.4–333.25)	26.27 (22.14–79.62)	17	0.195	0 (0–6.52)	0 (0–0)	27	0.437	34	<**0.001**
**Sesquiterpenes**										
α–Copaene	92.03 (55.42–127.64)	20.04 (19.15–32.94)	15	0.130	5.99 (0–274.28)	0 (0–54.56)	28	0.494	99	0.077
β–Elemene	302.68 (218.52–470.54)	81.68 (66.38–153.07)	14	0.104	0 (0–0)	0 (0–135.2)	30	0.616	42	<**0.001**
α–Gurjunene	25014 (13867–43497)	10832.43(4578.79–17090)	19	0.279	4421.58 (2181–8536)	3612.63 (3323.3–3920.47)	32	0.750	40	<**0.001**
*(E)*–β–Carryophyllene	2849.44 (312.8–13085.9)	3462.07 (246.9–34246)	30	1.000	1708.17 (395.5–4561.61)	2949.3 (678.5–7051.26)	33	0.820	136	0.590
Calarene	835.78 (705.89–1346.21)	383.35 (192.12–461.23)	16	0.160	4513 (1360.9–6797.7)	3601.3 (3396.4–4394.5)	35	0.964	36	<**0.001**
Azulene	304.37 (243.11–552.93)	173.62 (115.02–243.86)	19	0.279	1322.64 (724.8–2949)	1361.23 (991.24–1419.86)	35	0.964	29	<**0.001**
α–Humulene	309.48 (168.4–815.49)	570.1 (127.38–940.65)	29	0.959	110.29 (56.94–346.56)	314.54 (273.16–415.27)	20	0.151	100	0.083
Aromadendrene	24198 (17171–30173)	12884.1 (6317–13949.3)	19	0.279	132763 (41354–284546)	163211(131661–19030)	32	0.750	20	<**0.001**
Ledene	483.46 (385.14–920.15)	281 (127.27–341.22)	18	0.234	871.38 (425.58–1687.63)	955.59 (844.12–1110.07)	32	0.750	89	0.035
Palustrol	27519 (23454–33607.97)	14541 (10143–19643)	16	0.160	195280 (99185–582350)	299182 (199018–323158)	34	0.892	13	<**0.001**
Ledol	4321.5 (3323.9–5455.57)	2265.3 (1886.3–3032.2)	15	0.130	29916 (14969–9445.3)	45284 (32691.6–46581.9)	33	0.820	6	<**0.001**
**Other Compounds**										
Bornyl–acetate	203.80 (73.02–1055.02)	36.32 (28.2–179.66)	16	0.160	149.01 (60.45–5717.61)	262.12 (203.4–441.08)	29	0.553	122.5	0.318
6–Methyl–5–hepten–2–one	124.6 (84.21–300.85)	49.02 (46.73–49.7)	5	**0.006**	229.09 (85.36–427.8)	178.56 (125.86–221.6)	32	0.750	108	0.143
Citronellyl acetate	430.01 (134.65–624.06)	66.25 (57.15–190.04)	16	0.160	4158.7 (1106.8–8645.23)	2829.34 (1212.3–3648.8)	32	0.750	27	<**0.001**
Geranyl–acetate	1188.61 (283.8–1770.4)	362.6 (187.17–365.29)	16	0.140	5011.7 (1436.9–23411.4)	6117.16 (3346.6–6612.4)	36	1.000	47	<**0.001**
**Total**	270097.42 (171639.05–393122.98)	111185.27 (90739.17–185954.08)	17	0.195	728679.93 (358990.6–1515698.74)	741312.18 (702466.33–905714.86)	35	0.964	31	<**0.001**

### Exogenous Emissions from Mountain Birch (MB)

**Figure 4:**
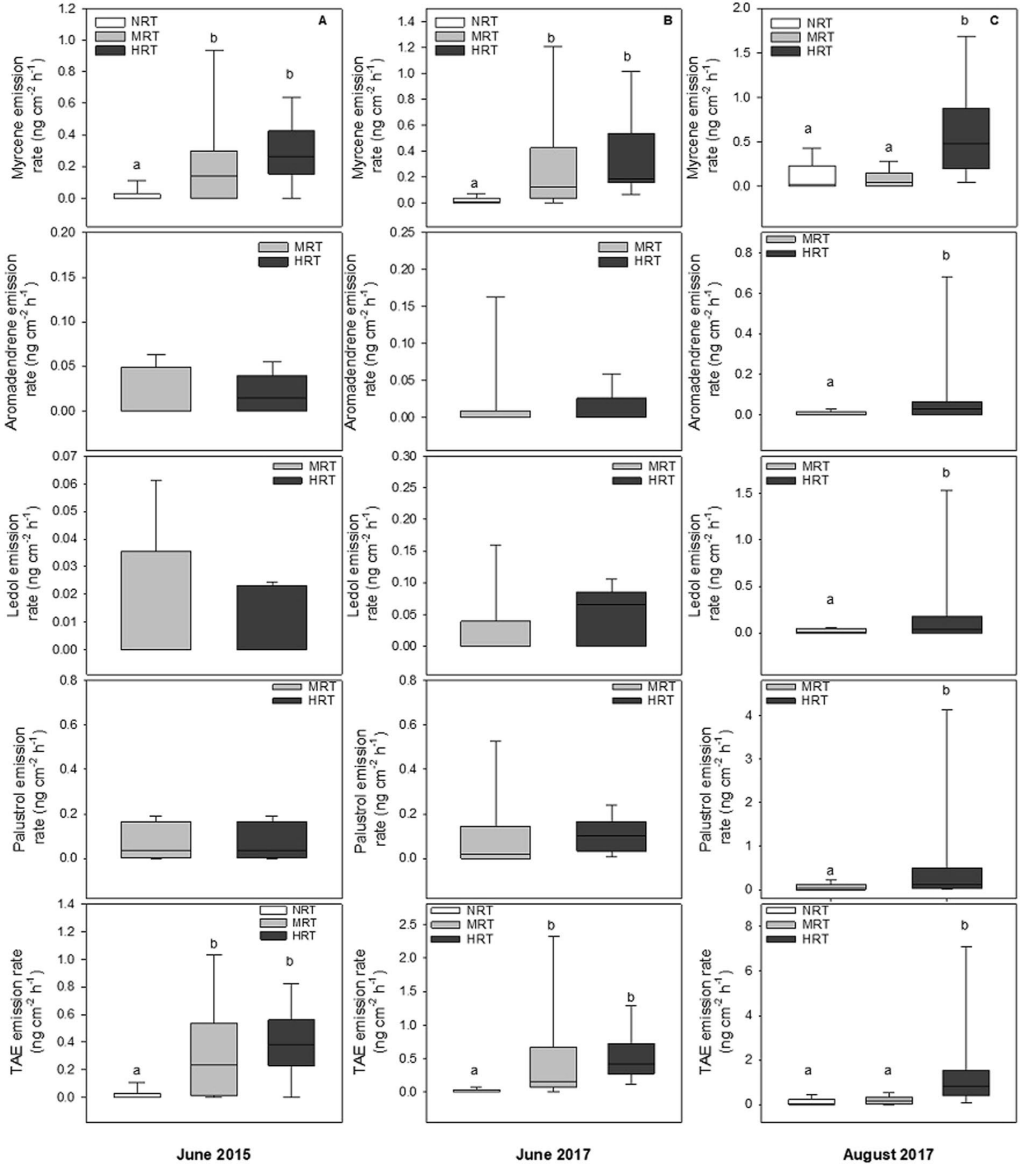
Emission rates (ng cm^−2^ h^−1^) of myrcene and recovered RT sesquiterpene compounds from mountain birch trees with no (NRT), moderate density (MRT) and high-density (HRT) *R*. *tomentosum* growing in the understorey. Box plots represent median and interquartile range with bars representing highest and minimum values. Differences in emission rates between groups were tested with Kruskal-Wallis non-parametric tests with multiple pairwise comparison (Mann-Whitney U with Bonferroni corrections). Aromadendrene, Palustrol and Ledol were recovered only in MRT and HRT plots and were tested with Mann-Whitney tests. Different letters above the bars indicate significant difference between groups (*P* < 0.05).

Myrcene, which is quantitatively the most abundant constituent of the RT volatile bouquet, was emitted at higher rates by MB in HRT group compared to other groups in all sampling occasions. In June 2015 χ^2^(2) = 6.828, N = 24, *P* = 0.039), June 2017 χ^2^(2) = 16.377, N = 24, *P* < 0.001) and in August 2017 χ^2^(2) = 15.545, N = 24, *P* < 0.001), Kruskal-Wallis H test (Fig. 4A–C). From multiple pairwise comparisons, MB in the HRT and MRT groups had higher myrcene emission rates than the NRT group in June 2015, and June 2017. In August, the HRT group’s myrcene emission rate was higher than the NRT and MRT groups (Fig. 4C).

The RT-specific compounds, ledol, and palustrol, as well as aromadendrene, were only recovered from MB trees in MRT and HRT groups. There was no significant difference between the MRT and HRT groups in early season (June 2017) samplings. In the late season (August 2017) sampling however, MB branches from HRT quadrants had higher emissions of palustrol and aromadendrene compared to MRT groups (Fig. 4A–C). The total adhered emissions (TAE) - recovered RT-sesquiterpenes and myrcene - was highest in HRT groups in June 2015 χ^2^(2) = 7.295, N = 24, *P* = 0.026) and June 2017 (χ^2^(2) = 20.746, N = 24, *P* < 0.001). In August 2017, TAE was higher in HRT than MRT and NRT (χ^2^(2) = 15.545, N = 24, *P* < 0.001), Kruskal-Wallis H test. TAE accounted for 0.5–0.8% of total MB emissions during early season sampling and up to 6.2% of total emissions in the late season sampling when MB trichome density had deteriorated and MB emission rates were low (Table S1).

### Early versus late season emissions

**Figure 5:**
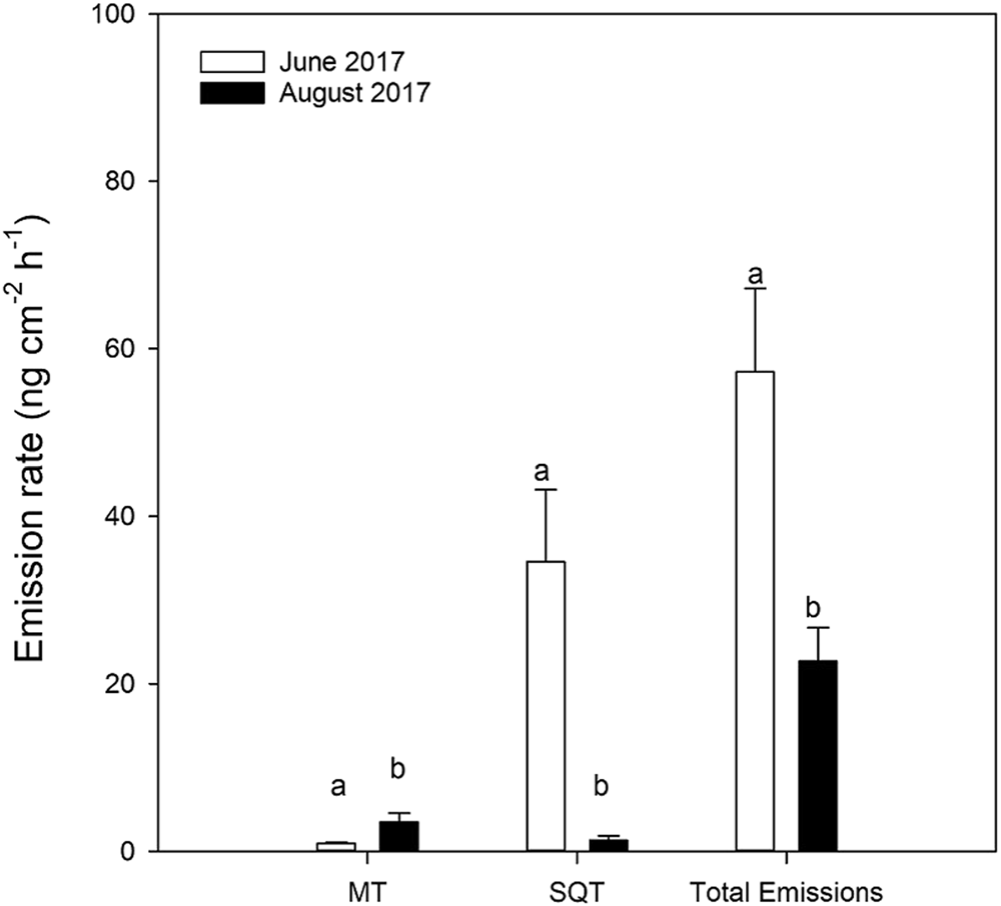
Total monoterpenes (MT), sesquiterpenes (SQT) and total emission rates from mountain birch (MB) trees in early season (June) and late season (August) 2017. Different letters^ab^ above bars indicate a significant difference between seasons (paired-sample t-test, *P* < 0.05).

MB monoterpene emission rates were higher in late season (August 2017) than in early season (June 2017), paired sample t-test, (*t*_(23)_ = −2.692, *P* < 0.001). This trend was reversed for sesquiterpene (*t*_(23)_ = 17.172, *P* < 0.001) and total VOC emission rates (*t*_(23)_ = 5.084, *P* < 0.001), paired sample t-test (Fig. 5). In RT, emission rates of most compounds were higher in late than early season (Table 1).

### Correlation between *R*. *tomentosum* abundance and recovery rate of adhered compounds

MB myrcene (r_s_ = 0.602, *P* = 0.008, N = 18) and ledol emission (r_s_ = 0.630, *P* = 0.001, N = 18) recovery rates correlated positively with the number of RT shoots growing in the understorey in June 2015 and June 2017 respectively. In August 2017, myrcene (r_s_ = 0.453, *P* = 0.059, N = 18), aromadendrene (r_s_ = 0.805, *P* < 0.001, N = 18), palustrol (r_s_ = 0.805, *P* < 0.001, N = 18), ledol (r_s_ = 0.460, *P* < 0.055, N = 18) and total adhered emission (TAE) (r_s_ = 0.579, *P* < 0.012, N = 18) rates correlated positively with the number of RT shoots in the understorey.

### Trichome assessment

**Figure 6:**
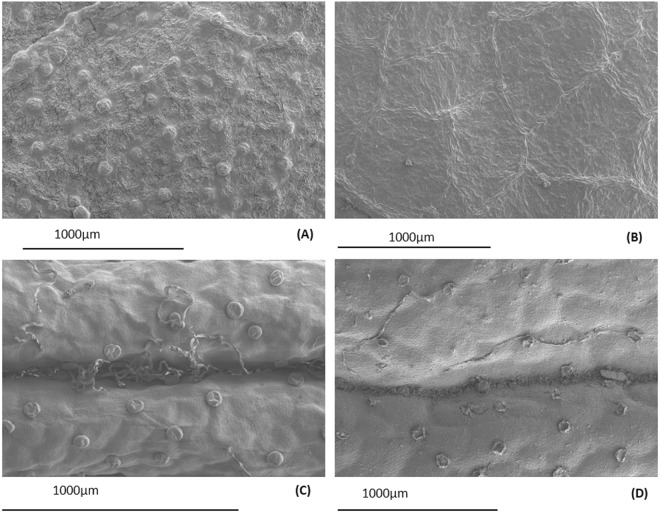
Scanning electron microscope (SEM) images of glandular trichomes on the lower side of mountain birch in (**A**) June and (**B**) August 2017, and intact (**C**) and deteriorated (**D**) glandular trichomes from *R*.*tomentosum* (RT) leaves.

The glandular trichome density on MB leaves was significantly higher in June than in August. Furthermore, the proportion of deteriorated glandular trichomes in August was higher than in June 2017 (Table 2). The upper side of the MB foliage had lower trichome density and a higher proportion of deteriorated trichomes (Table 2, Fig. 6A,B). For RT, the density of the trichomes on the adaxial leaf surface or stem were similar in leaf or stem age classes within the season (Table 3). The majority of glandular trichomes on leaves that developed in 2016 and 2015 were deteriorated, whereas those that had developed in 2017, and were sampled in August, were mainly intact (Table 3, Fig. 6C,D). Glandular trichome density in the stems did not change with age class or sampling date (Table 3). In general, the percentage of deteriorated trichomes was considerably lower on the stems than the adaxial surface of leaves (Table 3). Long, hairy trichomes were visibly more abundant in young than old stems (Fig. 1d,f).

**Table 2:** Median (Interquartile range) for glandular trichome density (# mm^−2^) and proportion (%) of deteriorated glandular trichomes on the upper and lower sides of Mountain birch (MB) leaves in June and August 2017. *P*-values and Mann-Whitney U values for differences between June and August are shown, *n* = 6.

Mean per leaf side	June	August	U	*P*-value
*Trichome density*				
Upper	6.8 (3.1–7.4)	1.2 (1–1.4)	3.0	0.030
Lower	13.1 (8.7–17.3)	2.9 (2.8–3.5)	0.0	0.002
*Deteriorated trichomes*				
Upper	0 (0–6.3)	31.5 (24.1–77.1)	0.5	0.004
Lower	0 (0–0.9)	8.1 (4.8–11.8)	4.5	0.026

**Table 3:** Mean (SE) values for glandular trichome density (# mm^−2^) and proportion (%) of deteriorated glandular trichomes on the adaxial surface of *Rhododendron tomentosum* (RT) leaves and stems in June and August 2017. n = 4. expect n = 2 for stem age classes 2015 and 2017 in August.

	June		August		
	Age class		Age class		
	2015	2016	2015	2016	2017
*Trichome density*					
Leaves	3.2 (0.7)	5.5 (0.6)	—	8.0 (1.2)	10.4 (1.2)
Stem	17.2 (2.5)	21.3 (2.2)	15.4 (4.5)	17.1 (1.8)	27. 9 (18.1)
*Deteriorated trichomes*					
Leaves	99.5 (0.49)	86.1 (7.8)	—	93.3 (2.4)	15.2 (11.7) 0
Stem	48.2 (24.4)	4.0 (1.8)	13.3 (6.7)	1.7 (1.1)	0 (0)

## Discussion

### *R. tomentosum* (RT) VOCs adhere to mountain birch (MB) foliage

Our results demonstrate the adherence and re-release of *R*. *tomentosum* (RT) VOCs by neighbouring mountain birch (MB) trees in the subarctic. This is in agreement with earlier reports by Himanen *et al*.^14,16^ where RT-specific sesquiterpenes were recovered from silver birch (*Betula pendula* Roth), downy birch (*Betula pubescens* Ehrh.) and broccoli, (*Brassica oleracea var*. *italica)* leaf surfaces. The sesquiterpene aromadendrene was in earlier reports emitted by *B*. *pubescens* in a peatland site with or without RT neighbours^14^, while this compound was adsorbed and re-emitted by *B*. *oleracea* exposed to RT neighbours for 24 hours at a temperature of 22 °C^16^.

Our results suggest that monoterpenes may also be adsorbed and re-emitted by neighbouring plants in subarctic ecosystems since myrcene, which is the major RT-monoterpene, was emitted at higher rates by MB trees growing above RT than the NRT group in our study. Myrcene was also emitted in minor quantities by MB trees growing with no RT understorey and has been shown to be synthesized and emitted by *Betula spp*^35,36^. Exogenous monoterpene fumigation experiments have resulted in monoterpene re-emission from exposed plant foliage^37,38^ in some cases for up to 12 hours post fumigation^38^. Field measurements in a boreal ecosystem have also showed there to be a slightly elevated emission of myrcene from *Betula spp*. growing in proximity with RT^14^. Myrcene is more volatile and reactive^28^ than the RT sesquiterpenes and is more readily re-emitted or oxidized in the atmosphere.

Leaf surface characteristics, air and leaf surface temperatures as well as physico-chemical properties of compounds are important factors in the determination of the deposition and re-release of VOCs from leaf surfaces^39^. Lipophilic uncharged VOCs such as terpenes tend to be adsorbed within the hydrophobic cuticular wax layer upon gas deposition^40^, from where they may be taken up into the leaf through the stomata^41,42^. Limonene uptake and re-release by 13 plant species was reported by Noe *et al*., (2008)^39^, with the uptake of the monoterpene scaling positively with leaf lipid content. Hydrophobic terpenes tend to partition in the leaf lipid phase^39,43^ which emphasizes the role of leaf lipid content in terpenoid adsorption. Stomatal uptake of terpenes from ambient air is also dependent on the concentration gradient of terpene in the air and the leaf boundary layer; when air concentrations are higher than the concentration within the leaf, there is uptake, while release occurs when ambient concentration becomes lower^40^. This bidirectional exchange of VOCs within vegetation and between vegetation and the atmosphere has been suggested as a possible reason for discrepancies in emission rates reported in different studies and for the presence of VOCs from anthropogenic sources to be found within a plant volatile bouquet^39^.

The effect of RT density on the recovery of RT VOCs from MB shoots was only evident during the late season sampling when the average sampling temperatures were 4 °C higher compared to the early season. This highlights the influence of temperature on VOC adsorption and re-emission^44^. Adsorption and re-emission of RT VOCs from neighbouring *B*. *oleracea* plants was higher when exposure was at a temperature of 22 °C than 6 °C^16^. Once released into the atmosphere, less volatile sesquiterpenoids may adhere to the surface of the emitting or neighbouring plants and condense on these surfaces during lower night-time temperatures, and may be re-released upon an increase in temperature^45^. Increase in temperature, combined with the emergence of new RT leaves with intact glandular trichomes during late season sampling, resulted in an increase in VOC emission rates. This contributed to the overall availability of these volatile and semi-volatile compounds for adsorption. The tendency of VOCs to be adsorbed by neighbouring plants is linked to the amount of volatiles available for adsorption^39,46^. The increase in VOC emissions from subarctic vegetation due to temperature changes^3,7,32^ combined with the temperature dependence of volatile adsorption and re-emission is likely to affect VOC emissions in these regions both quantitatively and qualitatively which in turn may affect the vegetation composition as well as atmospheric conditions in the region^47–49^.

### Mountain Birch (MB) and *R. tomentosum* (RT) emissions are related to trichome abundance

VOC emission rates from MB and RT were linked to leaf glandular trichome density and condition. Emergence of new leaves occurs early in the growing season for deciduous species like *Betula* and later in the season in evergreen species like RT^50,51^. In MB, early season sampling revealed a higher total VOC as well as sesquiterpene emission rate compared to late season sampling. The density of glandular trichomes on MB leaves was also higher in the early season. Glandular trichomes serve as storage pools for plant secondary compounds and emerge during the early stages of leaf development^4^. The final number of glandular trichomes in MB leaves is usually established during the early phase of leaf development and as such glandular trichome density is reduced drastically over time due to leaf expansion as well as natural deterioration^19^. The density of deteriorated glandular trichomes in late season was more than 10 times that of early season in mountain birch. Monoterpene emission rates in our study showed the reverse trend, with low emission rates in early season and increased emission rates in August. In comparison, the positive correlation between trichome density and VOC emissions in *B*. *nana* was only observed for sesquiterpene emissions and not monoterpene emissions^23^. The differences in MB monoterpene emission rates may also be due to temporal in-season variations as has been observed in MB trees growing in boreal ecosystems^52^. The variation in monoterpene emission rates was marked by a decrease in linalool emission rate and an increase in emission rates of sabinene and trans-ocimene^52^ from early to late season, similar trends were observed in our study (Table S1).

In RT, the emergence of new leaves with abundant and mainly intact glandular trichomes prior to the late season sampling coincided with an increase in total RT emissions that is in agreement with earlier work; thus revealing that essential oils of young RT shoots contained larger quantities of terpenoids than aged shoots^24^. The RT stems are also potentially important sources of VOCs, since they were covered with glandular trichomes that did not deteriorate between the two sampling dates and remained mainly intact in the previous year whorl. The 4 °C increase in mean sampling temperature between early and late season sampling may also have contributed to the increased emission rates, since de novo and storage pool emissions of terpenes are temperature dependent^4,32,39^.

MB terpenoid emission rate was higher in the NRT group than the MRT or HRT groups in both early and late season sampling. Our observation may be indicative of individual variation in VOC chemical quality and quantity that has been reported for MB trees^6,52,53^. The differences in understorey species composition (Table S2) of the different sites may also be indicators of factors like soil nutrient, microclimate or water availability, which may in turn affect VOC synthesis and emission^54,55^. The differences in species composition of the understorey^2,33^ may result in exposure of tree stands growing above to different blends of VOCs.

In summary, our study provides evidence for adherence and re-release of neighbouring plant VOCs in the subarctic; we showed an understorey RT density effect on the adsorption and re-emission of RT volatiles from neighbouring plant foliage. The high proportion of adhered RT compounds on MB, 6% of total BVOC emission of MB in August, was unexpected, but may be explained by the differences in the ontogeny of glandular trichomes in studied plant species. The alteration of the MB VOC emission blend by RT volatiles may have significant impact on volatile mediated interactions in MB especially in late season. The emission of VOCs by both plant species was linked to the presence of intact glandular trichomes during the sampling period. Altogether, our results provide evidence of a passive plant-to-plant interaction in subarctic ecosystems. Neighbouring plant VOC interference should therefore be considered in field measurements of VOCs.

## Electronic supplementary material

Dataset 1

## Data Availability

The datasets generated during and/or analysed during the current study are available from the corresponding author on reasonable request.
