# Supplementary material for: Understorey Rhododendron tomentosum and Leaf Trichome Density Affect Mountain Birch VOC Emissions in the Subarctic
Source: Sci Rep. 2018 Sep 5;8:13261. doi: 10.1038/s41598-018-31084-3 (PMC6125604; doi:10.1038/s41598-018-31084-3)
Supplement: Supplementary file 1 — Dataset 1 [file 41598_2018_31084_MOESM1_ESM.docx]

**Supplementary Data**

**S1**: Mean volatile emission rate (ng cm^-2^ h^-1^) of individual and total BVOC compounds from three mountain birch groups (No, moderate and high-density *R. tomentosum* plots) from three sampling periods.

| **Compounds** | **June 15** | | | **June 17** | | | **August 17** | | |
| --- | --- | --- | --- | --- | --- | --- | --- | --- | --- |
|  | NRT  (n=6) | MRT  (n=12) | HRT  (n=6) | NRT  (n=12) | MRT  (n=24) | HRT  (n=12) | NRT  (n=12) | MRT  (n=24) | HRT  (n=12) |
| **Monoterpenes** |  |  |  |  |  |  |  |  |  |
| α-Thujone | 0.00 | 0.00 | 0.00 | 0.00 | 0.01 | 0.01 | 0.02 | 0.03 | 0.01 |
| α-Pinene | 1.13 | 0.86 | 0.23 | 0.72 | 0.20 | 0.17 | 0.91 | 0.94 | 0.16 |
| Camphene | 0.04 | 0.01 | - | 0.04 | 0.00 | 0.01 | 0.05 | 0.04 | - |
| Sabinene | 0.36 | 1.10 | 0.00 | 0.00 | 0.00 | 0.00 | 1.42 | 2.19 | 0.02 |
| β-pinene | 0.20 | 0.13 | 0.02 | 0.04 | 0.00 | 0.00 | 0.22 | 0.24 | 0.02 |
| Δ-3-Carene | 0.14 | 0.09 | 0.13 | 0.02 | 0.00 | 0.02 | 0.00 | 0.01 | 0.00 |
| Limonene | 1.10 | 1.01 | 0.42 | 0.24 | 0.15 | 0.14 | 0.49 | 0.49 | 0.13 |
| 1.8 Cineole | 0.00 | 0.01 | 0.02 | 0.00 | 0.00 | 0.00 | 0.02 | 0.08 | 0.04 |
| β-Ocimene | 0.72 | 0.13 | 0.03 | 0.09 | 0.04 | 0.28 | 0.61 | 0.34 | 0.84 |
| γ-Terpinene | 0.00 | 0.06 | 0.00 | 0.00 | 0.05 | 0.00 | 0.05 | 0.06 | 0.00 |
| Linalool | 0.72 | 0.32 | 0.12 | 0.18 | 0.14 | 0.58 | 0.02 | 0.02 | 0.00 |
| Total Monoterpenes | 4.40 | 3.72 | 0.95 | 1.33 | 0.61 | 1.21 | 3.80 | 4.45 | 1.22 |
| **Sesquiterpenes** |  |  |  |  |  |  |  |  |  |
| α-Copaene | 0.61 | 0.37 | 0.24 | 2.80 | 1.06 | 2.54 | 0.10 | 0.00 | 0.00 |
| (E)-β-Caryophyllene | 5.78 | 7.11 | 1.36 | 49.52 | 7.31 | 7.97 | 1.81 | 0.64 | 0.21 |
| α-Humulene | 0.39 | 0.83 | 0.21 | 7.82 | 1.44 | 1.98 | 0.34 | 0.12 | 0.00 |
| Germacrene | 2.12 | 0.47 | 0.20 | 2.20 | 0.30 | 1.46 | 0.08 | 0.01 | 0.10 |
| Caryophyllene oxide | 4.01 | 3.19 | 1.80 | 11.90 | 6.38 | 15.00 | 0.21 | 0.40 | 0.03 |
| α-Cubenene | - | - | - | 0.25 | 0.01 | 0.02 | - | - | - |
| Iso-Caryophyllene | - | - | - | 0.76 | 0.06 | 0.05 | - | - | - |
| β-Selinene | - | - | - | 0.45 | 0.07 | 0.22 | - | - | - |
| Total Sesquiterpenes | 12.91 | 11.97 | 3.80 | 75.72 | 16.62 | 29.24 | 2.54 | 1.17 | 0.34 |
| **RT Compounds** |  |  |  |  |  |  |  |  |  |
| Myrcene | 0.02 | 0.22 | 0.29 | 0.02 | 0.40 | 0.41 | 0.12 | 0.10 | 0.54 |
| Aromadendrene | - | 0.02 | 0.02 | - | 0.03 | 0.01 | - | 0.01 | 0.13 |
| Palustrol | - | 0.07 | 0.08 | - | 0.13 | 0.12 | - | 0.08 | 0.62 |
| Ledol | - | 0.02 | 0.01 | - | 0.03 | 0.05 | - | 0.03 | 0.21 |
| α-Gurjunene | - | - | - | - | 0.02 | 0.00 | - | 0.00 | 0.00 |
| Total adhered emission | 0.02 | 0.33 | 0.40 | 0.02 | 0.61 | 0.58 | 0.12 | 0.21 | 1.48 |
| **GLVs** |  |  |  |  |  |  |  |  |  |
| (E)-2-Hexenal | 0.47 | 1.08 | 0.50 | 0.30 | 0.44 | 0.56 | 0.35 | 0.27 | 0.12 |
| (Z)-3-Hex-1-ol | 18.47 | 10.52 | 7.16 | 4.80 | 5.15 | 11.94 | 8.58 | 3.50 | 3.85 |
| (Z)-2-hexen-1-ol | 0.53 | 0.31 | 0.54 | 0.10 | 0.69 | 1.22 | 0.32 | 0.05 | 0.09 |
| 1-Hexanol | 0.54 | 0.49 | 0.26 | 0.05 | 0.19 | 1.16 | 0.10 | 0.10 | 0.11 |
| (Z)-3-hex-acaetate | 20.07 | 17.79 | 7.77 | 2.06 | 3.78 | 15.60 | 12.27 | 6.64 | 5.03 |
| Nonanal | 2.94 | 3.19 | 1.71 | 5.42 | 5.30 | 3.51 | 1.67 | 2.60 | 1.97 |
| (Z)-3-hex-butyrate | 3.12 | 2.97 | 1.47 | 0.81 | 1.04 | 1.80 | 0.69 | 0.18 | 0.34 |
| (Z)-3-hex-isovalerate | 0.69 | 0.34 | 0.15 | 0.00 | 0.06 | 0.19 | 0.49 | 0.24 | 0.15 |
| Total GLVs | 46.84 | 36.71 | 19.55 | 13.54 | 16.65 | 35.97 | 24.47 | 13.60 | 11.65 |
| **Other Compounds** |  |  |  |  |  |  |  |  |  |
| 6-Methyl-5-hept-2-one | 0.22 | 0.21 | 0.12 | 0.40 | 0.61 | 0.46 | 0.07 | 0.13 | 0.09 |
| (E)-DMNT | 0.40 | 0.03 | 0.00 | - | - | - | 1.42 | 0.13 | 0.15 |
| MeSA | 0.55 | 0.07 | 0.00 | - | - | - | 0.26 | 0.05 | 0.03 |
| Phenol | 0.14 | 0.36 | 0.13 | - | - | - | - | - | - |
| Acetic acid, hexyl ester | 1.02 | 1.03 | 0.20 | - | - | - | - | - | - |
| Butanoic acid 2-hexenyl ester | 0.31 | 0.52 | 0.10 | - | - | - | - | - | - |
| 4-Methylene-2,8,8- trimethyl-2-  vinylbicyclo(5.2.0) nonane | 4.14 | 5.03 | 2.95 | - | - | - | - | - | - |
| **Total Emissions** | 70.94 | 60.00 | 28.20 | 91.01 | 35.11 | 67.41 | 36.10 | 19.73 | 15.16 |

-Not detected

**(S2)** – Vegetation analysis of 1m^2^ plots of understorey of sampled mountain birch (MB) trees including distance between VOC sampling branch and *Rhododendron tomentosum* (RT) shrubs. Average values ± SE represented (n=6 for control and high RT plots and 12 for moderate *Rt* density plots).

| **Treatment** | **Non RT** | | **Moderate RT** | | **High RT** | |
| --- | --- | --- | --- | --- | --- | --- |
|  | **2015** | **2017** | **2015** | **2017** | **2015** | **2017** |
| **Tree Height (m)**  **Plant Densities**  RT Shoots  RT flowering shoots  **MB branch – RT shoot distance***  **Plant Coverage (%) ****  **Angiosperms**  *Rhododendron tomentosum*  *Vaccinium vitis-idea*  *Vaccinium myrtilus*  *Empetrum nigrum*  *Linnea borealis*  *Vaccinium uliginosum*  *Pyrola sp*  **Club mosses (Lycopodiopsida)**  *Lycopodium annotinum*  **Mosses**  *Pleurozium schreberi*  *Hylocomium splendens*  **Lichens**  *Cladonia rangiferina*  *Cladonia arbuscula*  *Peltigera apthosa*  *Cetraria nivalis* | 3.5 ± 0.4^a^  -  -  105.7 ± 7  0  30 ± 8.7  6.7 ± 2^a^  35 ± 8.8 ^ab^  5.2 ± 3.1  2.5 ± 1.7 ^ab^  -  -  65 ± 8.5^a^  14.2 ± 9.3  1.2 ± 0.6  1 ± 0.5  2.2 ± 1.6  1.7 ± 1.7 | 4.1 ± 0.4  -  -  101.2 ± 6.3  0  25 ± 7.6  5.2 ± 2.4  32.5 ± 7.9^a^  0.8 ± 0.4  -  -  -  67.5 ± 7.5^a^  9.2 ± 6.4  -  2 ± 1.1  1.3 ± 0.8  1.2 ± 1.2 | 4.1 ± 0.3^ab^  20.3 ± 2.6^a^  0.2 ± 0.2  105.9 ± 5.4  14.1 ± 2  17.1 ± 3.1  4.8 ± 1.6^ab^  45.4 ± 7.1^a^  4.0 ± 1.1  11.2 ± 2.9 ^a^  -  0.1 ± 0.1  21.7 ± 4.9^b^  31.4 ± 6.9  1.8 ± 1.2  0.6 ± 0.3  0.5 ± 0.4  0.9 ± 0.5 | 4.7 ± 0.5  25.3 ± 3.5^a^  0.2 ± 0.2  94.7 ± 6.1  12.2 ± 2.3  18.3 ± 2.7  11.8 ± 3.4  34.6 ± 5.1^a^  1.8 ± 0.8  6.1 ± 2.2  -  -  28.8 ± 5.5^b^  27.9 ± 5.6   - 1. ± 0.08   0.9 ± 0.5  -  1.2 ± 0.5 | 5.3^b^ ± 0.17  85.8 ± 9.4^b^  2.0 ± 0.9  100.2 ± 9.2  60 ± 5.3  20 ± 7.4  1.2 ± 0.8^b^  18 ± 5.3 ^b^  6.5 ± 3.1  0.7 ± 0.4 ^b^  -  -  22.5 ± 6.7^b^  13.7 ± 3.1  -  -  -  - | 5.9 ± 0.8  72.5 ± 13.06^b^  2.2 ± 1  93.6 ± 7.9  51.7 ± 11.9  29.2 ± 11.6  3.7 ± 2.4  11.2 ± 2.5^b^  2.7 ± 0.8  0.8 ± 0.4  0.3 ± 0.2  -  24.2 ± 8.7^b^  13.7 ± 3.8  -  -  -  0.3 ± 0.3 |

*Distance from tip of sampled MB branch to base of RT shoots 2015 and tip of RT shoots (or understorey vegetation in control) in 2017.

**Total leaf area coverage can exceed 100% of the soil area as leaves of different plant species are in different layers.

^ab^ represents significant difference (Kruskal-Wallis test) between treatment groups for each year.

- Not present.
